# Cytostatic activity of oxidized tetracycline in vitro: relevance for the treatment of malignant effusions?

**DOI:** 10.1038/bjc.1988.117

**Published:** 1988-05

**Authors:** C. Sauter

**Affiliations:** Department of Medicine, University Hospital, ZÃ¼rich, Switzerland.

## Abstract

**Images:**


Br. J. Cancer (1988), 57, 514–515 © The Macmillan Press Ltd., 1988

SHORT COMMUNICATION

### Cytostatic activity of oxidized tetracycline *in vitro*: relevance for the treatment of malignant effusions?

C. Sauter

*Division of Oncology, Department of Medicine, University Hospital, CH-8091 Zürich, Switzerland.*

Malignant pleural effusions are frequently treated today by instillation of tetracycline (Hausheer & Yarbro, 1985). The hypothesis that the low pH of the tetracycline solution is the reason for the therapeutic benefit (Sahn & Good, 1979) could not be substantiated by a randomized study comparing tetracycline with an acified multivitamin solution of similar pH and aspect (Zaloznik *et al.*, 1983). In recent experiments we demonstrated cytostatic activity of decomposed tetracycline and suggested that oxidation of the tetracycline molecule is necessary for this cytostatic action (Sauter & Cogoli, 1987). The present experiments were designed to study the cytostatic activity of the three main degradation products of tetracycline, i.e. anhydrotetracycline, 4-epi-tetracycline, and 4-epi-anhydrotetracycline.

Tetracycline HCl, anhydrotetracycline HCl, 4-epi-tetracycline HCl, and 4-epi-anhydrotetracycline HCl were obtained from American Cyanamid Company, Lederle Laboratories Division, USA. Heating of tetracycline HCl (Sauter & Cogoli, 1987) was done after dissolving the drug (500 mg of tetracycline in 10 ml distilled water).

A human hypernephroma line (Groscurth & Kistler, 1977) was used as previously described (Sauter *et al.*, 1986).

For inhibition of cell growth human hypernephroma cells were incubated with the different tetracycline molecules in the following way (Sauter & Cogoli, 1987): To 1 ml of medium (RPMI 1640 supplemented with 8% foetal bovine serum) containing a given drug concentration 1 ml of cell suspension with ~120,000 hypernephroma cells freshly prepared by trypsinization of a cell monolayer was added. This mixture was incubated for 15 min at 37°C in sterile 7 ml screw-capped centrifuge tubes. After incubation the cells were centrifuged at 200 g for 10 min at 20°C, and then resuspended in 4.2 ml of fresh medium; 1.0 ml of the cell suspension was put in each of 4 wells of sterile flat bottom plastic plates (24 wells, diameter 16 mm, Costar, Cambridge, Mass., USA). The plates were then incubated at 37°C in a 5% $CO_2$ atmosphere. After 4 days of incubation – at this time the control wells (only medium without drugs) showed a complete monolayer containing $\sim 1.3 \times 10^6$ cells per well – the cells were stained by methylenblue/parafuchsin (Kistler & Bischof, 1962) and the plates evaluated. In the case of continuous drug contact to 1 ml of the original cell suspension (~120,000 cells), 1 ml of medium and 2 ml of drug dilution were added, thoroughly mixed, and also distributed into 4 wells. There was no change of medium until the end of the experiment after four days.

The evaluation of the stained plates was done over a neon screen with a photographic light meter (Lunsix 3, Gossen, FRG) containing an adapter piece fitting the 16 mm wells of the plastic plates. The results were recorded the following way: Complete growth inhibition (Figure 1f) corresponded to a light intensity (measured in lux) equal to that of plates stained two hours after cell seeding. Detectable inhibition of growth (Figure 1b, e) corresponded to an increase of light intensity of at least 25% in each well over the control wells.

Table 1 shows the concentrations necessary to produce growth inhibition of hypernephroma cells by tetracycline either prepared freshly or heated for 10 min at 100°C, and by the three main degradation products. All of them cause a
b
c
d
e
f

**Figure 1** Growth inhibition by different tetracycline derivatives (concentration 22 $\mu$M); continuous exposure of a hypernephroma cell line. a: Medium control: complete monolayer. b: Tetracycline HCl, freshly prepared: detectable inhibition. c: Tetracycline HCl, heated for 10 min at 100°C: almost complete inhibition. d: Anhydrotetracycline HCl: almost complete inhibition. e: 4-epi-tetracycline HCl: detectable inhibition. f: 4-epi-anhydrotetracycline HCl: complete inhibition.

**Table I** Growth inhibition (GI) by tetracycline HCl, heated tetracycline HCl, and three derivatives; continuous exposure of a hypernephroma cell line

| *Drug* | *Minimal concentration* ($\mu$*M*) *for:* complete GI | *detectable GI* |
|---|---|---|
| Tetracycline HCl | 44 | 22 |
| Tetracycline HCl (100°C/10 min) | 44 | 11 |
| Anhydrotetracycline HCl (AT) | 44 | 11 |
| 4-epi-tetracycline HCl | 44 | 22 |
| 4-epi-anhydrotetracycline HCl (4-EAT) | 22 | 11 |

Received 8 December 1987; and in revised form, 26 February 1988.

growth inhibition at similar concentrations. The heated tetracycline and the two oxidized molecules (anhydrotetracycline and 4-epi-anhydrotetracycline) are only slightly more active during continuous drug-cell contact. Figure 1 demonstrates this difference: at a concentration of 22 $\mu$M freshly prepared tetracycline (b) and 4-epi-tetracycline (e) produce only detectable growth inhibition whereas the heated tetracycline and the anhydrotetracycline produce almost complete and the 4-epi-anhydrotetracycline complete inhibition of growth. At the same time Figure 1 illustrates the definitions of complete and detectable inhibition of growth as described. Table II shows that the non-oxidized molecules have no cytostatic activity up to concentrations of 112 $\mu$M during short time drug-cell contact.

Different drugs and techniques have been proposed for the treatment of malignant effusions. Instillation of cytotoxic drugs like bleomycin or nitrogen mustard is effective as well as tube drainage and treatment with tetracycline (Hausheer & Yarbro, 1985). The effect of tetracycline is ascribed to its sclerosing effect (Sahn & Good, 1979). A study by Zaloznik *et al* (1983) however, suggests an additional mechanism of action since tetracycline treatment was more successful than a sclerosing procedure alone. The present experiments demonstrate that tetracycline – at least in its oxidized form – is cytostatic. In the tetracycline vial (Achromycine®) ascorbic acid is always added to prevent oxidation. In the pleural cavity or by heating this protection of tetracycline by ascorbic acid is rapidly lost. Oxidation products (anhydrotetracycline {AT} and 4-epi-anhydrotetracycline {4-EAT}) appear already after a few hours of incubation (Thomson *et al.*, 1984). The estimated concentrations of AT and 4-EAT in the pleural cavity after the usual instillation of 1,000 mg tetracycline HCl must be at least 100 times higher than the concentration required for complete growth inhibition of tumour cells *in vitro* (see Table II).

**Table II** Growth inhibition by tetracycline HCl, heated tetracycline, and three derivatives: concentration 112 $\mu$M; drug-cell contact 15 minutes

| *Drug* | *Growth inhibition* |
|---|---|
| Tetracycline HCl, fresh | none |
| Tetracycline HCl (100°C/10 min) | complete |
| Anhydrotetracycline HCl (AT) | complete |
| 4-epi-tetracycline HCl | none |
| 4-epi-anhydrotetracycline HCl (4-EAT) | detectable |

The mechanism of cytostatic action of AT and 4-EAT can presently only be a matter of speculation. AT and 4-EAT resemble the anthracyclines in their structure being of a biosynthetic origin similar to the tetracyclines (Hutchinson, 1981). Tetracycline binds to DNA (Kohn, 1961) and could, therefore, act like anthracyclines as an intercalating agent.

The next obvious step will be to determine AT and 4-EAT concentrations in pleural fluid and serum after tetracycline instillation. Serum analysis may give an indication of possible side effects known to be produced by degraded tetracyclines (Frimpter *et al.*, 1963). These determinations will certainly be a prerequisite for the treatment of malignant effusions by AT and 4-EAT. According to the *in vitro* results these substances should be more efficient than the original tetracycline molecule.

In conclusion, the therapeutic value of the tetracycline treatments of malignant effusions may be due to a double effect of tetracycline HCl, *viz.* a cytostatic *and* a sclerosing action.

This work was supported by the 'Schweizerische Krebsliga'. I thank Ms H. Ernst, Ms. Ch. Meier, and Ms. L. Resenterra for technical assistance and Mrs. E. Sauter for linguistic help.

### References

FRIMPTER, G.W., TIMPANELLI, A.E., EISENMENGER, W.J., STEIN, H.S. & EHRLICH, L.I. (1963). Reversible 'Fanconi syndrome' caused by degraded tetracycline. *J. Am. Med. Assn.*, **184**, 111.

GROSCURTH, P. & KISTLER, G.S. (1977). Human renal cell carcinoma in the nude mouse: long term observations. *Beitr. Pathol.*, **160**, 337.

HAUSHEER, F.H. & YARBRO, J.W. (1985). Diagnosis and treatment of malignant pleural effusion. *Sem. Oncol.*, **12**, 54.

HUTCHINSON, C.R. (1981). The biosynthesis of tetracycline and anthracycline antibiotics. In *Antibiotics*, Corcoran, J.W. (ed) Vol. IV, p.1. Springer: Berlin.

KISTLER, G.S. & BISCHOFF, A. (1962). Zur exfoliativen Zytologie kleiner Flüssigkeitsmengen. *Schweiz. med. Wschr.*, **92**, 863.

KOHN, K.W. (1961). Mediation of divalent metal ions in the binding of tetracycline to macromolecules. *Nature*, **191**, 1156.

SAHN, S.A. & GOOD, J.T. (1979). The pH of sclerosing agents. *Chest*, **76**, 198.

SAUTER, Chr., COGOLI, M. & ARRENBRECHT, S. (1986). Interactions of cytotoxic and other drugs: rapid cell culture assay. *Oncology*, **43**, 46.

SAUTER, CHR. & COGOLI, M. (1987). Tetracycline in the treatment of malignant effusions: evidence for a cytostatic action of the decomposed drug. *Eur. J. Cancer Clin. Oncol.*, **23**, 973.

THOMSON, H.J., MERANI, S. & MILLER, S.S. (1984). Storage of tetracycline solutions for peritoneal lavage. *J. Royal Coll. Surg. Edin.*, **29**, 379.

ZALOZNIK, A.J., OXWALD, S.G. & LANGIN, M. (1983). Intrapleural tetracycline in malignant pleural effusions. A randomized study. *Cancer*, **51**, 752.


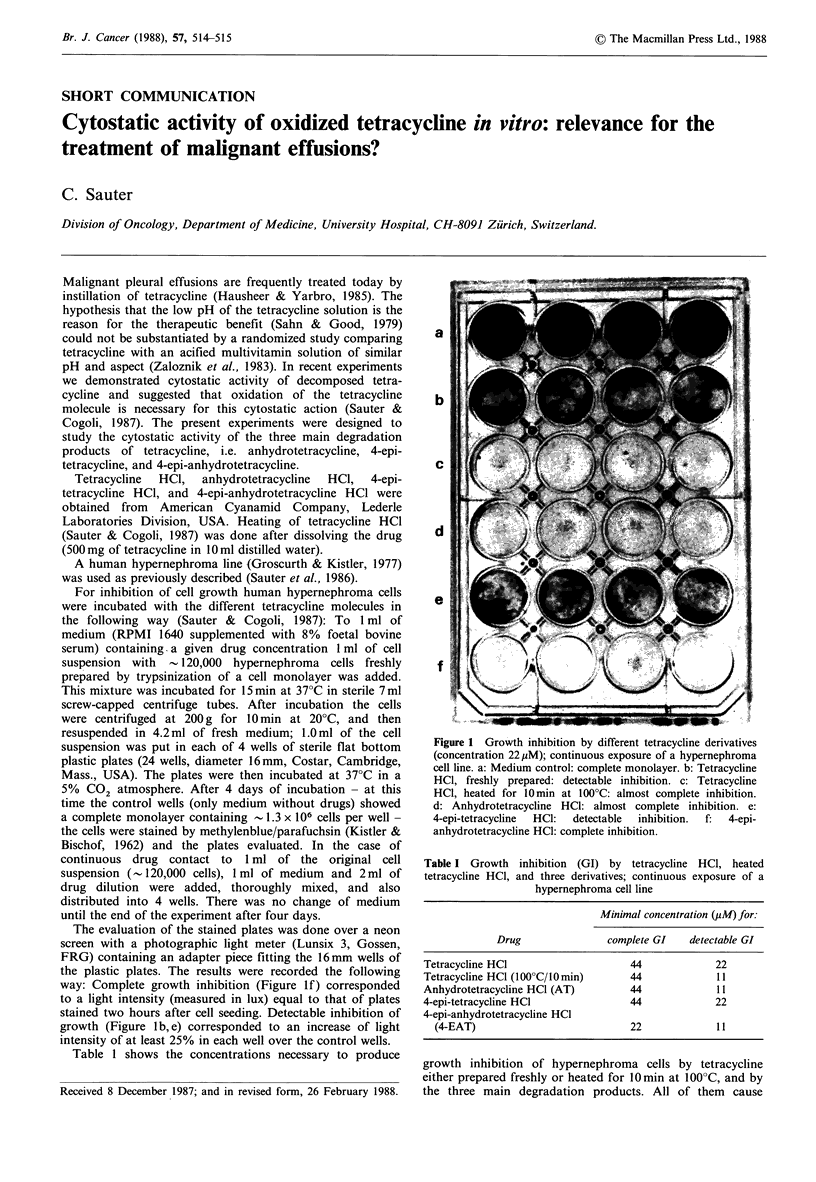

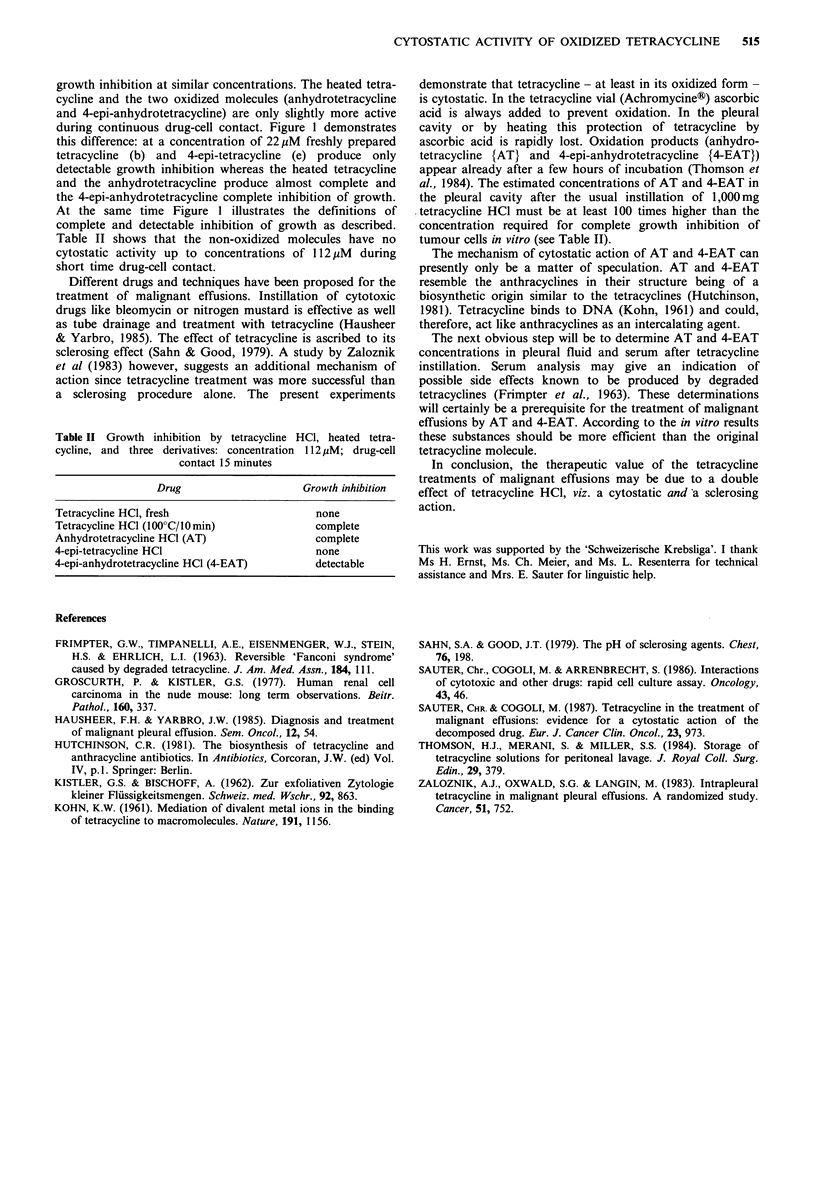


## References

[OCR_00201] FRIMPTER G. W., TIMPANELLI A. E., EISENMENGER WJ STEIN H. S., EHRLICH L. I. (1963). Reversible "Faconi syndrome" caused by degraded tetracycline.. JAMA.

[OCR_00206] Groscurth P., Kistler G. (1977). Langzeitbeobachtungen an menschlichen hypernephroiden Nierenkarzinomen in der "nude" Maus.. Beitr Pathol.

[OCR_00211] Hausheer F. H., Yarbro J. W. (1985). Diagnosis and treatment of malignant pleural effusion.. Semin Oncol.

[OCR_00220] KISTLER G. S., BISCHOFF A. (1962). [On exfoliative cytology of small quantities of fluids].. Schweiz Med Wochenschr.

[OCR_00224] KOHN K. W. (1961). Mediation of divalent metal ions in the binding of tetracycline to macromolecules.. Nature.

[OCR_00228] Sahn S. A., Good J. T., Potts D. E. (1979). The pH of sclerosing agents: a determinant of pleural symphysis.. Chest.

[OCR_00237] Sauter C., Cogoli M. (1987). Tetracycline in the treatment of malignant effusions: evidence for a cytostatic action of the decomposed drug.. Eur J Cancer Clin Oncol.

[OCR_00242] Thomson H. J., Merani S., Miller S. S. (1984). Storage of tetracycline solutions for peritoneal lavage.. J R Coll Surg Edinb.

[OCR_00247] Zaloznik A. J., Oswald S. G., Langin M. (1983). Intrapleural tetracycline in malignant pleural effusions. A randomized study.. Cancer.

